# The EU project Real4Reg: unlocking real-world data with AI

**DOI:** 10.1186/s12961-025-01287-y

**Published:** 2025-02-27

**Authors:** Jonas Peltner, Cornelia Becker, Julia Wicherski, Silja Wortberg, Mohamed Aborageh, Inês Costa, Vera Ehrenstein, Joana Fernandes, Steffen Heß, Erzsébet Horváth-Puhó, Monika Roberta Korcinska Handest, Manuel Lentzen, Peggy Maguire, Niels Henrik Meedom, Rebecca Moore, Vanessa Moore, Dávid Nagy, Hillary McNamara, Anne Paakinaho, Kerstin Pfeifer, Liisa Pylkkänen, Blair Rajamaki, Evy Reviers, Christoph Röthlein, Martin Russek, Célia Silva, Dirk De Valck, Thuan Vo, Elvira Bräuner, Holger Fröhlich, Cláudia Furtado, Sirpa Hartikainen, Aleksi Kallio, Anna-Maija Tolppanen, Britta Haenisch

**Affiliations:** 1 German Center for Neurodegenerative Diseases (DZNE), Bonn, Germany; 2 Research Division, Federal Institute for Drugs and Medical Devices, Kurt-Georg-Kiesinger-Allee 3, 53175 Bonn, Germany; 3 Department of Bioinformatics, Fraunhofer Institute for Algorithms and Scientific Computing (SCAI), Sankt Augustin, Germany; 4 INFARMED, National Authority of Medicines and Health Products, I.P., Health Technology Assessment Department (DATS), Lisbon, Portugal; 5 Department of Clinical Medicine, Department of Clinical Epidemiology, and Center for Population Medicine, Aarhus University, Aarhus, Denmark; 6 INFARMED, National Authority of Medicines and Health Products, I.P., Information and Strategic Planning Department (DIPE), Lisbon, Portugal; 7 Health Data Lab (FDZ), Federal Institute for Drugs and Medical Devices, Bonn, Germany; 8 Data Analytics Center, Danish Medicines Agency, Copenhagen, Denmark; 9 European Institute for Women’s Health (EIWH), Dublin, Ireland; 10 School of Pharmacy, University of Eastern Finland, Kuopio, Finland; 11 Department of Oncology, University of Turku, Turku, Finland; 12 European Association for Professionals and People with ALS (EUpALS), Louvain, Belgium; 13 Bonn-Aachen International Center for IT, University of Bonn, Bonn, Germany; 14 NOVA National School of Public Health, NOVA University Lisbon, Lisbon, Portugal; 15 CSC-IT Center for Science Ltd., Espoo, Finland; 16 Center for Translational Medicine, Medical Faculty, University of Bonn, Bonn, Germany

## Abstract

**Background:**

The use of real-world data is established in post-authorization regulatory processes such as pharmacovigilance of drugs and medical devices, but is still frequently challenged in the pre-authorization phase of medicinal products. In addition, the use of real-world data, even in post-authorization steps, is constrained by the availability and heterogeneity of real-world data and by challenges in analysing data from different settings and sources. Moreover, there are emerging opportunities in the use of artificial intelligence in healthcare research, but also a lack of knowledge on its appropriate application to heterogeneous real-world data sources to increase evidentiary value in the regulatory decision-making and health technology assessment context.

**Methods:**

The Real4Reg project aims to enable the use of real-world data by developing user-friendly solutions for the data analytical needs of health regulatory and health technology assessment bodies across the European Union. These include artificial intelligence algorithms for the effective analysis of real-world data in regulatory decision-making and health technology assessment. The project aims to investigate the value of real-world data from different sources to generate high-quality, accessible, population-based information relevant along the product life cycle. A total of four use cases are used to provide good practice examples for analyses of real-world data for the evaluation and pre-authorization stage, the improvement of methods for external validity in observational data, for post-authorization safety studies and comparative effectiveness using real-world data. This position paper introduces the objectives and structure of the Real4Reg project and discusses its important role in the context of existing European projects focussing on real-world data.

**Discussion:**

Real4Reg focusses on the identification and description of benefits and risks of new and optimized methods in real-world data analysis including aspects of safety, effectiveness, interoperability, appropriateness, accessibility, comparative value creation and sustainability. The project’s results will support better decision-making about medicines and benefit patients’ health.

*Trial registration* Real4Reg is registered in the HMA-EMA Catalogues of real-world data sources and studies (EU PAS number EUPAS105544).

## Background

The European Union (EU)’s healthcare systems aim for equitable access to effective medicines, supported by fast, data-driven regulatory decisions and health technology assessment (HTA). Randomized controlled trials (RCT), the gold standard study design to support regulatory decision-making, typically have high internal validity and are designed to assess efficacy. However, effectiveness, that is, benefits in real-life scenarios, may be different due to variation in patients, clinical settings and duration and adherence to treatments. To address limitations of clinical trials, pharmaceutical companies and regulators use real-world data (RWD) for evidence generation, complementing traditional RCTs. RWD is defined by the European Medicines Agency (EMA) as “routinely collected data relating to a patient’s health status or the delivery of health care from a variety of sources other than traditional clinical trials” [1]. Real-world evidence (RWE) derived from RWD is integral in all drug development phases and the authorization process [2–4]. RWE can complement traditional RCTs by overcoming some of the limitations of RCTs [5, 6]. RCTs for example, are usually conducted in relatively homogeneous study populations which differ from the real-world populations, which will later use the medication in terms of socio-demographic characteristics such as age, sex or socioeconomic status [7, 8]. Moreover, RCTs cannot answer all important research questions for healthcare decision-makers due to ethical, financial or resource reasons; impeding the conduct of RCTs and RWE can be used in the pre-authorization to overcome those limitations and complement RCTs [9–12].

Today, RWE is predominantly used in post-authorization for monitoring safety signals and identifying unique sub-populations. However, although RWE has a promising role to inform regulatory decision-making, use of RWD is often constrained by challenges with respect to data access, by challenges in analysing data from different settings and sources and by following best practices in design and analyses [1, 13, 14]. There are currently no evidentiary standards for the use of RWE in regulatory and HTA advise and decision-making. Regulatory agencies such as the EMA or the U.S. Food and Drug Administration and other stakeholders have, however, recently started publishing frameworks, guidance and guidelines on the use of RWD and RWE in regulatory decision-making [15–17]. In the field of academia, initiatives such as the Joint ISPE-ISPOR Special Task Force in on Real‐World Evidence in Health Care Decision Making have published good practices and recommendations on how to generate RWE from RWD [18, 19], and target trial emulation [20–22] was established as an important framework for comparative effectiveness studies using RWD.

Given the increased use of RWD and RWE, regulators and HTA bodies need to be able to validate claims made using these data through independent analyses and to independently assess the evidentiary value of RWD and RWE. To enable the use of RWD and RWE in the context of regulatory decision-making and HTA, the EU recently provided funding to six projects within the European Community’s Horizon Europe Programme (ID HORIZON-HLTH-2022-TOOL-11-02). One of these projects is Real4Reg (“Use cases for development, optimization and implementation of artificial intelligence methods for real-world data analyses in regulatory decision-making and health technology assessment along the product lifecycle”; www.real4reg.eu), which aims to develop, optimize and implement artificial intelligence (AI) methods for RWD analyses in regulatory decision-making and HTA assessment along the product lifecycle. AI is a sub-field of computer science that focusses on the development of systems displaying intelligent behaviour. Machine learning (ML) is a sub-field of AI dealing with the development of statistical models that can infer patterns from data and generalize to unseen data, thus being able to perform certain tasks without explicit instruction [23]. In Real4Reg, different ML techniques will be applied. These include generative algorithms to create synthetic patient trajectories, algorithms to derive RWD-based external control arms, algorithms to derive propensity scores used to mitigate confounding in RWD studies and different techniques to estimate average treatment effects and conditional average treatment effects. Furthermore, ML methods will be used to cluster patients on the basis of their medical history. This position paper introduces the Real4Reg project, its structure and objectives, and discusses its role in the context of existing European projects and initiatives focussing on RWD.

## Design

### Objectives and tasks

Real4Reg started in January 2023 and has a 48-month duration. The project is funded by the European Commission within the Horizon Europe Framework Programme (grant agreement 101095353) and is part of the MetReal cluster in which six projects collaborate to promote synergies. In the MetReal cluster, Real4Reg is unique as it spans the entire spectrum from highly relevant use cases for RWE from regulatory and HTA practice across the product lifecycle. The Real4Reg consortium consists of 10 partners from 6 European countries with experience in the field of RWD analyses. Experts from regulatory agencies and HTA bodies, academia and patient organizations are part of the consortium. The project has three overall objectives. It aims to enable the use of RWD and RWE by developing user-friendly data-driven tools and technologies for the effective analyses of RWD in regulatory decision-making and HTA. To establish the value of RWD and RWE on regulatory decision-making and HTA, Real4Reg will integrate the methods developed as part of the first objective into tool packages to provide scientists within health regulatory and HTA bodies with ready-to-use methodological standards. The results will inform training activities on good practice examples and will be informative for existing and emerging guidelines for both health regulatory authorities and HTA bodies across Europe.

Real4Reg is registered in the HMA-EMA Catalogues of real-world data sources and studies (EU PAS number EUPAS105544) and detailed study protocols, which describe the planned analyses and studies developed and published in the course of this registration. Real4Reg has been awarded the ENCePP Seal, which is awarded to studies in the HMA-EMA Catalogues of real-world data sources and studies that uphold high standards in pharmacoepidemiology and pharmacovigilance research, ensuring scientific independence, transparency and robust methodologies, thereby minimizing potential biases and avoiding conflicts of interest.

### Use cases

**Fig. 1:**
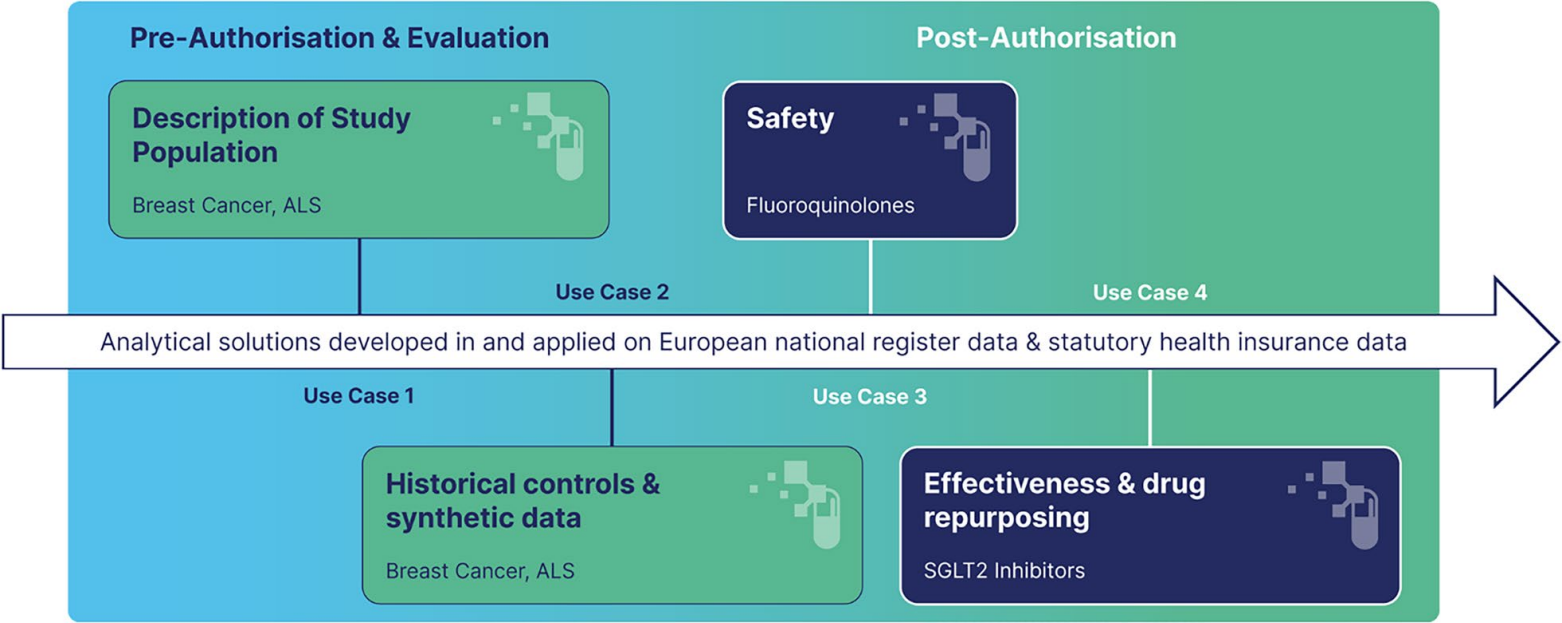
The four Real4Reg use cases

**Fig. 2:**
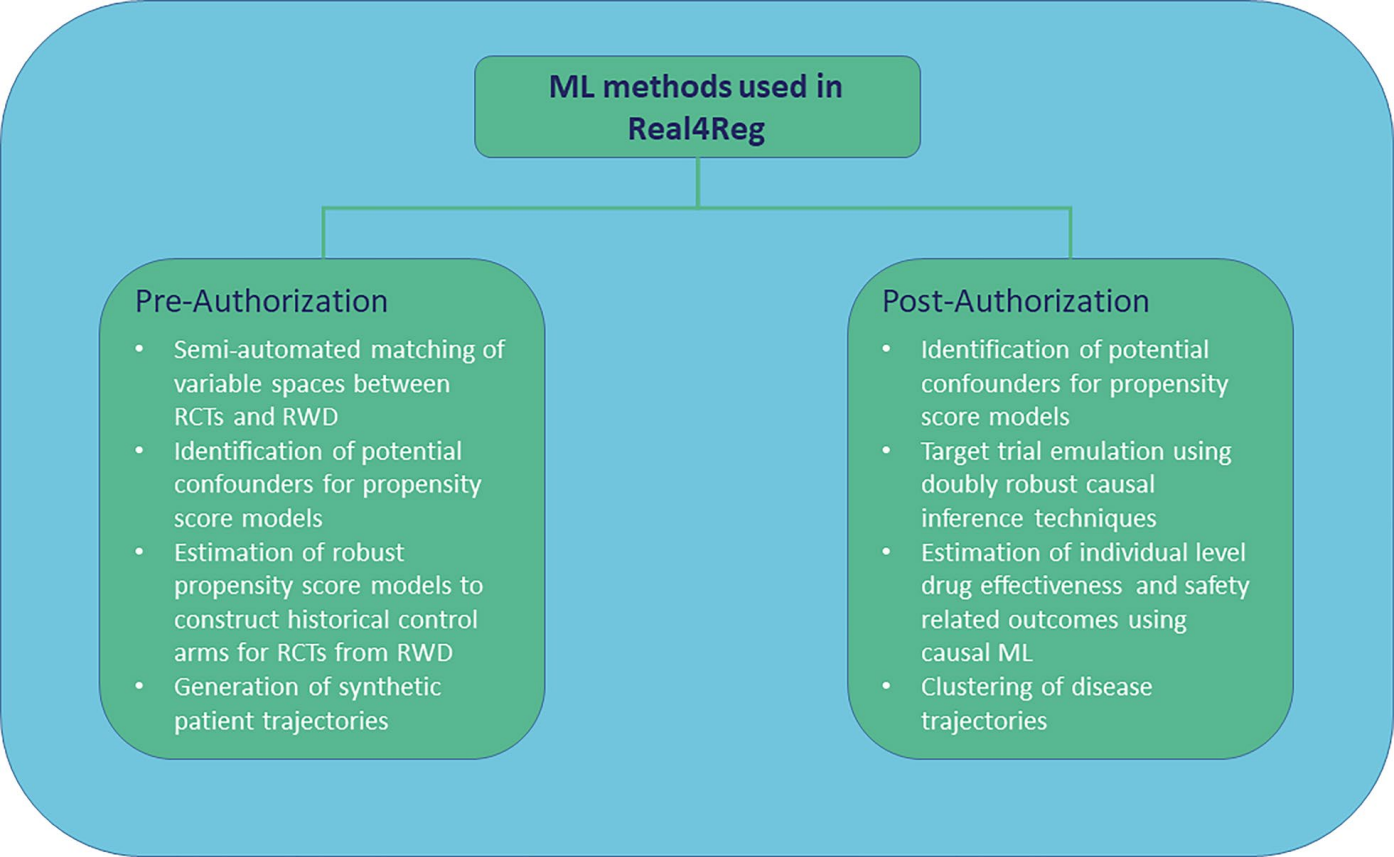
Machine Learning methods used in Real4Reg

The overarching aim of Real4Reg is to investigate the value of RWD from European health and administrative databases to generate high-quality, accessible, population-based information relevant along the product life cycle. Therefore, as shown in Fig. 1, the objective of use cases (UCs) 1 and 2 is the preparation of good practice examples for analyses of RWD for the evaluation and pre-authorization stage and the improvement of methods for external validity in observational data. UCs 3 and 4 will prepare good practice examples for post-authorization safety studies and comparative effectiveness on the basis of RWD. Study protocols and information on the methods and techniques applied in the different use cases are published in the HMA-EMA catalogues of real-world data sources and studies [24]. Figure 2 gives an overview of the different purposes for which ML methods will be used in the project.

Use case 1 focusses on breast cancer (BC) and amyotrophic lateral sclerosis (ALS), representing common and rare diseases, respectively. Use case 1 showcases data sources’ heterogeneity stemming from differences in coding systems and practices; data provenance and the associated biases; underlying population, completeness and temporal trends; and risk of bias related to how data are captured and reported. In addition, completeness is investigated and described. Natural history of both diseases, as well as their incidence, prevalence, mortality, survival time, signals of disease progression, and changes in standards of care will be described. Workflows that allow for selection of patients in real-world datasets, display of patient trajectories and assessment of patient characteristics of patients with BC and ALS will be provided.

Use case 2 investigates the application of historical control arms on the basis of RWD and explores whether generative AI approaches could be used to counterfactually simulate for individuals observed in the real-world possible outcomes in a RCT. ML techniques will be used to estimate robust propensity score models to construct historical control arms for patients who participated in ALS trials using RWD. Multimodal neural ordinary differential equations [25] will be used to generate synthetic data of patients with ALS included in the RWD data sources. These data will subsequently be used to simulate possible future disease trajectories of those patients. In both applications ML techniques will also be used to semi-automatically map variables in the RCT data sources to their equivalents in the RWD data sources. Furthermore, UC2 includes descriptions of the treatment of BC in populations neglected in RCTs such as pregnant women, women with comorbidities such as psychiatric conditions or cardiovascular diseases that may influence participation in RCTs, women aged 65 years and older and men with BC.

Use case 3 assesses the risk of pre-specified adverse drug reactions (ADRs) and evaluates the impact of regulatory warnings on the use of fluoroquinolones (FQs). A descriptive drug utilization study will illustrate changes in prescription retrievals of FQs and other broad-spectrum antibiotics and user characteristics. The risk of ADRs associated with FQ use compared with other broad-spectrum antibiotics will be assessed in a cohort study using an active comparator new user (ACNU) design. Incidences of ADRs, hazard ratios, absolute risk differences and number needed to harm will be estimated. Potential confounders to be included in the analyses will be identified using propensity scores estimated with different ML techniques. Furthermore, doubly robust causal inference methods will be used to estimate the influence of using FQs on the future risk of adverse events conditional on patient specific covariates such as age, sex, past medications and diagnoses. To assess individual level safety-related outcomes, causal ML methods will be used [26, 27]. Explainable AI techniques such as Shapley additive explanations (SHAP) [28] will be used to interpret trained ML models and to identify potential biases.

Use case 4 focusses on effectiveness and drug repurposing. In a study using an ACNU design, the effectiveness of sodium-glucose linked transporter 2 (SGLT-2) inhibitors to prevent overall and cause-specific hospitalization and mortality is evaluated using dipeptidyl peptidase-4 (DPP-4) inhibitors as an active comparator. National time trends in the use of new non-insulin anti-diabetics introduced in the study period will also be reported. ML models trained to predict the effectiveness of SGLT-2 inhibitors for individual patients will allow for the generation of further hypotheses about whether patients with diagnoses other than the indication of the medicine might benefit from treatment. For this purpose, the same ML techniques as in UC3 will be used. Clustering algorithms will be applied to identify groups of patients with similar disease trajectories.

### Data and data sources

The data used for the project include health and administrative databases from four European countries. All data sources have been utilized for pharmacoepidemiological and clinical epidemiology analyses. The validity of all data sources has been reviewed [29–37]. The data cover adult populations of Denmark, Finland, Germany and Portugal. Table 1 lists the data sources, estimated sizes of the source populations and available variables for each of the four data-providing countries.

**Table 1:** Key characteristics of the data sources used in Real4Reg

Country	Type of data	Size of source population	Items
Denmark	National healthcare register data	5.9 million	Administrative and health data on procedures, diagnoses, drug dispensing, cause of death, laboratory results, socio-demographic data
Finland	National healthcare register data	5.6 million	Administrative and health data on procedures, diagnoses, drug dispensing, cause of death, laboratory results, socio-demographic data
Germany	Claims data	72.8 million	Billing data on procedures, diagnoses, drug prescriptions and drug dispensing and socio-demographic data
Portugal	National healthcare register data	10.3 million	Administrative and health data on procedures, diagnoses, drug dispensing, socio-demographic data

Real4Reg aims to enable the use of different RWD in a standardized way. To do so, all study data are converted to a common data model. The common data model used in Real4Reg will be a common data model derived from the Observational Medical Outcomes Partnership (OMOP) [38]. The Real4Reg common data model represents a subset of the OMOP tables and variables, which are of relevance for the use cases in this project.

### Contextualization, dissemination, exploitation and communication

Real4Reg aspires to proactively engage RWE-related stakeholders for interaction and critical reflection. An advisory board consisting of patients and clinical experts as well as from industry, regulatory and HTA bodies involved with RWE will provide an overall perspective of and advice on the project. Our outreach activities include a common data model and a library of analytical workflows from the UCs and guidance and training concepts for the use of RWE in regulatory decision-making and HTA, as well as expert work meetings, RWE workshops (including patient symposia), scientific publications and a public domain webpage (www.real4reg.eu) informing about the plans, current state and progresses of the project. Additionally, press releases and social media platforms such as LinkedIn will be used to engage the general public and share relevant progress and news concerning the Real4Reg project. The library of analytical workflows and other software generated in the project will be published openly on a dedicated GitLab repository. In compliance with local regulations and to uphold and preserve privacy, individual-level data will remain at their respective data custodians in their native countries. Metadata will be published when legally permitted on the Real4Reg project web page and in a Zenodo repository. Results will be shared with the scientific community via regular open-access publications, contributions to scientific conferences, regular workshops and symposia, updates to the project websites and a newsletter. The exchange with other stakeholders will be facilitated by bi-annual advisory board meetings, the involvement of patient advocacy groups as consortia partners and close connections to regulatory bodies such as EMA and healthcare organizations (such as the WHO) and relevant committees working on the implementation of long-term plans to connect pharmacological research with RWE.

## Discussion

In recent years, EMA and other EU regulatory agencies have been investing in better access to RWD and have been working on establishing the value of RWE in regulatory decision-making. The use of RWD and RWE for development and use of better medicines is envisioned by the European regulatory network and is anchored in the EMA network strategy to 2025 [39, 40]. The EMA network strategy identifies several challenges to increase the use of RWD and RWE. Those include limitations in access to and sharing of RWD, a lack of required competencies in areas such as AI and ML, data science and advanced analytics, and a lack of regulatory standards, guidance and validation of RWD and RWE. Real4Reg contributes to the efforts made by the EMA and addresses some of the challenges together with other projects of the MetReal Cluster and initiatives involved in establishing the European Health Data Space (EHDS), including DARWIN EU and TEHDAS [41, 42]. The project’s main purpose is to optimize and support the work of regulatory authorities, HTA bodies and academia by helping them to incorporate evidence generated from RWD in their processes. The overall objectives of the project include enabling, establishing and impacting the use of RWD and RWE through the development and provision of data analytical solutions, usable standards and guidance and training.

To enable the use of RWD and RWE in a standardized way, Real4Reg will provide health regulatory and HTA bodies with new and optimized workflows that enable the assessment and application of the effective use of RWD, RWE and synthetic data in all phases along the product lifecycle. These will include software packages of analytical and ML methods on the basis of good practice examples. Using the provided tools, health regulatory and HTA bodies will be able to optimize study designs of RCTs to predict and assess drug repurposing parameters and to streamline and improve monitoring of drug safety and effectiveness. Furthermore, by implementing an OMOP-derived common data model, Real4Reg aims to provide a better understanding of the technological challenges that arise when different datasets are harmonized and standardized for subsequent analyses. This takes up one recommendation from a recent EMA review on RWE use in regulatory decision-making, which calls for use of more diverse RWD data sources and combination of data sources from different European countries [40, 43]. Real4Reg aims to provide impulses to enable and enhance the use of ML techniques with RWD in a regulatory context. This includes optimization of existing and development of new ML methods for study population selection, summary statistics, construction of synthetic control arms and standardized results reporting, as well as for clustering of disease trajectories and risk profiles. In a reflection paper under consultation, EMA explicitly embraces the use of AI in the medicinal product lifecycle [43]. On technical aspects, it stipulates tasks for which Real4Reg is developing or optimizing ML-based methods. These include, for example, exploratory data analyses to describe the data characteristics, representativeness and relevance for the intended task or synthetic data generation. Furthermore, the EMA reflection paper highlights the need for identification of potential biases in AI models. Real4Reg will provide workflows and guidance to detect and avoid different sources of biases in ML models used to evaluate RWD. Moreover, the EMA calls for applying ethical principles for AI to the phases of the product lifecycle, such as human oversight and transparency, which is addressed by making study protocols publicly available and following the ENCePP standards. Although Real4Reg will highlight why transparency in reporting in reporting strengths and weaknesses of data sources and AI models is of utmost importance, the way in which the recently passed EU AI Act will affect the applicability of the project’s result is at the moment not clear. The EU AI Act establishes a common regulatory and legal framework for AI in the European Union and covers all types of AI in a broad range of sectors. AI used for research purposes is excluded, which means that the research on AI models planned in the project is not affected by the new law. It could, however, influence practical implementation AI-based data-driven methods and tools for the assessment of medicinal products developed in the project. To ensure that the developed methods can be applied by regulatory and HTA bodies, the project will follow the guidance on the use of AI in the medicine lifecycle, which will be published by the EMA in 2024.

Although RWE is increasingly used in post-authorization processes [3, 44], its value for regulatory decision-making is still a subject of debate [5]. In Real4Reg, we aim to establish the value of RWD and RWE by providing guidance concepts on data-driven regulatory decision-making and HTA to address current needs for guidance and validation and encounter some of the key challenges still existing when utilizing RWD [1, 15, 40, 45]. The guidance provided by Real4Reg will help health regulatory and HTA bodies to better assess and critically discuss strengths and limitations of RWD studies. It will include good practice examples on RWD use in pre-authorization, evaluation and post-authorization steps of the product lifecycle and also have recommendations on definitions for usable standards in data quality, data analyses and reporting. The guidance will not only be informative to health regulatory and HTA bodies or the regulatory science community, but will also inform payers and policy-makers about the value of RWE and how to use it in legislative and policy contexts. Lastly, the guidance informs patients about the use and evidentiary value of RWD. It will highlight how patients can make an impact on improving public health by proactively sharing health data in a self-determined and secure way.

On the basis of the workflows and methods developed during the project as well as on the guidance concepts, Real4Reg will provide health regulatory and HTA bodies with a blended learning training concept for data-driven decision-making using emerging data types. The practically oriented training concept aims to change the perception of the opportunities for the use of RWD and RWE in regulatory decision-making and HTA. It will demonstrate the value of RWE in conjunction with data from RCTs and how the potential of RWD in the regulatory context can be exploited and used effectively in a knowledge-enhancing way for the development of new medicines.

## Conclusions

The development of new and improved methods and technologies is a key driver of innovation in healthcare. Real4Reg focusses on the management of benefits and risks of new and optimized methods in RWD considering aspects of safety, efficacy, effectiveness, interoperability, appropriateness, accessibility, comparative value creation and sustainability. The project’s results will support decision-making about medicines and ultimately benefit patients’ health.

## Data Availability

No datasets were generated or analysed during the current study.

## Abbreviations

ACNU: Active comparator new user
ADR: Adverse drug reaction
AI: Artificial intelligence
ALS: Amyotrophic lateral sclerosis
BC: Breast cancer
DPP-4: Dipeptidyl peptidase-4
EHDS: European Health Data Space
EMA: European Medicines Agency
ENCePP: European Network of Centres for Pharmacoepidemiology and Pharmacovigilance
EU: European Union
FQ: Fluoroquinolone
HTA: Health technology assessment
ISPE: International Society for Pharmacoepidemiology
ISPOR: Professional Society for Health Economics and Outcomes Research
ML: Machine learning
OMOP: Observational Medical Outcomes Partnership
RCT: Randomized controlled trials
RWD: Real-world data
RWE: Real-world evidence
SGLT-2: Sodium-glucose linked transporter 2
UC: Use case
